# Effectiveness and safety of electroacupuncture in treating Parkinson disease: A protocol for systematic review and meta-analyses

**DOI:** 10.1097/MD.0000000000025095

**Published:** 2021-03-12

**Authors:** Wei Xu, Sha OuYang, Zhenhai Chi, ZhiQin Wang, DaoCheng Zhu, RiXin Chen, GenPing Zhong, FengTing Zhang, GuiQin Zhou, SiWei Duan, Lin Jiao

**Affiliations:** a The Affiliated Hospital of Jiangxi University of Traditional Chinese Medicine, Nanchang, China; b Science and Technology college of Jiangxi University of Traditional Chinese Medicine.

**Keywords:** electroacupuncture, parkinson disease, randomized controlled trial, systematic review

## Abstract

**Background:**

Parkinson disease (PD) is an Extrapyramidal Disease mainly characterized by static tremor, myotonia, bradykinesia and postural gait disorder. As China's population ages, the number of people suffering from PD is increasing. Since there are many side effects of western medicine for Parkinson's patients, and the high price of the drugs make it difficult for many patients to adhere to treat. At present, many clinical studies have shown that electroacupuncture is effective in treating PD. Therefore, this systematic review aims to explore the effectiveness and safety of electroacupuncture in the treatment of PD.

**Methods:**

Comprehensive search of PubMed, Embase, Medline, Cochrane Database of Systematic Reviews, Chinese Biomedical Literatures Database, China National Knowledge Infrastructure, Chinese Scientific Journal Database, Wang Fang Database from inception to February 2021, the literature selected is not restricted by language. In addition, we will search for unpublished studies and the references that were originally included in the literature manually. There were two reviewers screened the data and cross-checked the information individually, the quality of the literature was assessed by reviewers independently. The outcomes of interest include:the scale of Unifified PD Rating Scales, the Webster scale, the Quality of Life Questionnaire, total effective rate, recurrence rate, adverse events. The laboratory inspection indicators include:the content of lipid peroxidase, Superoxide dismutase activity in plasma and erythrocyte. The relevant randomized controlled trials will be included in this study. And we will evaluate the quality of the selected literature according to the Cochrane Handbook. Meta-analysis will be performed using RevMan 5.4.0 software. The heterogeneity test will be implemented in the included literature, the tests’ thresholds will be *P* < .1 and *I*^2^ > 50%. We will use either fixed effects model or random effects model according to the size of heterogeneity.

**Results:**

The results of this systematic review will provide a comprehensive evidence for the clinical treatment of PD, and we will report this result soon.

**Conclusion:**

This paper will explore whether or not electroacupuncture can be used as a non-drug therapy for PD.

**Ethics and dissemination:**

Ethical approval is not required for this paper, our plan will be published in the journal.

**Trial registration number:**

INPLASY202120031

## 1 Introduction

Parkinson disease (PD) is an Extrapyramidal Disease mainly characterized by static tremor, myotonia, bradykinesia and postural gait disorder. At the same time, PD is accompanied by a large number of non-motor symptoms, such as hyposmia, constipation, depression, sleep disorders, etc.^[1]^ This disease occurs most often in the elderly, the prevalence of PD in people over 65 years old was about 1% to 2%, this proportion in the people over 85 years old accounted for 3 to 5 percent.^[2,3]^ As China's population ages, the number of people suffering from PD is increasing, which puts great economic pressure on families and society. At present, the etiology and pathogenesis of PD are not clear, it may be related to the factor of environment, age and heredity.^[4]^ There have been reports that the main pathological features of PD may include many respects, such as the degeneration and necrosis of dopaminergic neurons, the formation of Lewybody and the decreasing of dopamine neurotransmitters.^[5,6]^

PD is mainly treated with oral drugs, such as Levodopa, this approach is to restore balance of nervous system function through exogenous supplementation that increases the level of dopamine in our body. However, the long-term use of this treatment will produce many adverse reactions, such as on-off phenomena and dyskinesia. These side effects mentioned above and the financial burden of long-term medication makes it difficult for patients to adhere to treat.^[7]^

Traditional Chinese medicine, including acupuncture, is effective in treating PD, and traditional Chinese medicine has the characteristics of simple, convenient, cheap and so on.^[8,9]^ Acupuncture can relieve the symptoms of PD patients and improve their quality of life by raising the level of dopamine in the blood, and reducing the amplitude and frequency of tremor muscle potentially.^[10,11]^

Electroacupuncture is one form of acupuncture therapies, it has a good effect on PD.^[12–14]^ But there is a lack of high-level evidence-based medical evidence to support this conclusion. Although there were systematic reviews have confirmed that acupuncture has a positive effect on PD.^[9]^ However, there is still a lack of systematic reviews on electroacupuncture treatment for PD, and this is urgently needed in the clinic. Therefore, this study will systematically review the effectiveness and safety of electroacupuncture in treating PD, in order to provide a more reliable evidence for the treatment of PD.

## 2 Methods and analysis

### 2.1 Study registration

This systematic review's plan has been registered on the platform, registration number: INPLASY202120031. You can check its authenticity on this website(https://inplasy.com/inplasy-2021–2-0031/). This article doesn’t require ethical approval, and only analyse the effectiveness and safety of electroacupuncture in treating PD that has been published in various databases. The protocol follows the Cochrane Handbook for Systematic Reviews and Meta-Analysis Protocol(PRISMA-P).^[15]^

### 2.2 Eligibility criteria

#### 2.2.1 Type of studies

We will comprehensively search the literature about electroacupuncture in treating PD in Chinese and English databases. In addition, unpublished documents will be searched manually. The non-randomized controlled trials must be excluded.

#### 2.2.2 Types of participants

Participants in this study were required to meet the international recognized diagnostic criteria for PD.^[16]^ There is no restriction on age, sex, nationality, but the PD participants will be excluded if one accompanied by serious cardiovascular and cerebrovascular diseases, and life-threatening complications.

#### 2.2.3 Types of interventions

PD participants in the test group must be treated with electroacupuncture as the main regimen (either in combination with other treatments or alone) and the control group must be treated with non-electroacupuncture therapy.

#### 2.2.4 Type of comparators

In the control group, the intervention means may include medicine(Traditional Chinese Medicine, western medicine), routine symptomatic treatment etc, which electroacupuncture should be used for the only requirement.

1. Electroacupuncture therapy vs no treatment;
2. Electroacupuncture therapy vs placebo;
3. Electroacupuncture therapy vs sham acupuncture;
4. Electroacupuncture therapy vs symptomatic or active treatment;

#### 2.2.5 Types of outcome measures

##### 2.2.5.1 Primary outcomes

The total effective rate and the total symptom score were the main outcomes . The total symptom score will be based on the scale of Unifified PD Rating Scales (including the score of spirit, behavior, emotion, daily activities, motor function, motor complications) and the Webster scale.

##### 2.2.5.2 Secondary outcomes

1. Quality of Life Questionnaire^[17]^;
2. The score of Hamilton Depression Scale;
3. Laboratory inspection indicators:the level of the content of lipid peroxidase, Superoxide dismutase activity in plasma and erythrocyte;
4. Recurrence rate;
5. Adverse events;

### 2.3 Exclusion criteria

Non-randomized controlled trial;Electroacupuncture is present in the control group; Repeated literature, theoretical discussion and review literature, nursing literature, animal experimental research, etc.

### 2.4 Search strategy

The following databases will be searched:PubMed, Embase, Medline, Cochrane Database of Systematic Reviews, Chinese Biomedical Literatures Database, China National Knowledge Infrastructure, Chinese Scientific Journal Database, Wang Fang Database from inception to February 2021. The main subject terms searched: “electroacupuncture,” “Parkinson,” “Parkinson disease,” “parkinsonism,” Pubmed's search strategy is shown in Table 1; Other databases’ search strategies will be adjusted according to each database.

**Table 1:** The search strategy for Pubmed.

Order	strategy
#1	Search “electroacupuncture”[Mesh]
#2	Search “electroacupuncture” [Ti/Ab]
#3	#1 OR #2
#4	Search “Parkinson disease”[Mesh] OR “”[Mesh] OR “parkinsonism”[Mesh] OR “Parkinson”[Mesh]
#5	Search “Parkinsonian Syndrome” [Ti/Ab] or “Parkinsonian Syndromes^∗^ ” [Ti/Ab] or “Parkinsonian Diseases”[Ti/Ab] or “Parkinsonism” [Ti/Ab] or “Autosomal Dominant Parkinsonism”[Ti/Ab] or “ Dominant Parkinsonism” [Ti/Ab]
#6	#4 AND #5
#7	Search “Randomized controlled trial” [MeSH] or “controlled clinical trial” [MeSH]
#8	Search “Randomized controlled trial” [Ti/Ab] or “clinical trial” [Ti/Ab] or “randomized” [Ti/Ab]
#9	#7OR #8
#10	#3 AND #6 AND #9

### 2.5 Process of selection

#### 2.5.1 Information screening

The included literature will be conducted as follows:First, imported the retrieved literature into NoteExpress 3.1software, and eliminate the duplicate literature in the document manager; Second, remove the literature not relevant to this study by reading the title and abstract of literature one by one; Third, download the remaining articles in sequence and read the full text; Finally, according to the inclusion and exclusion criteria required in this article, then the final paper is determined.

**Figure 1:**
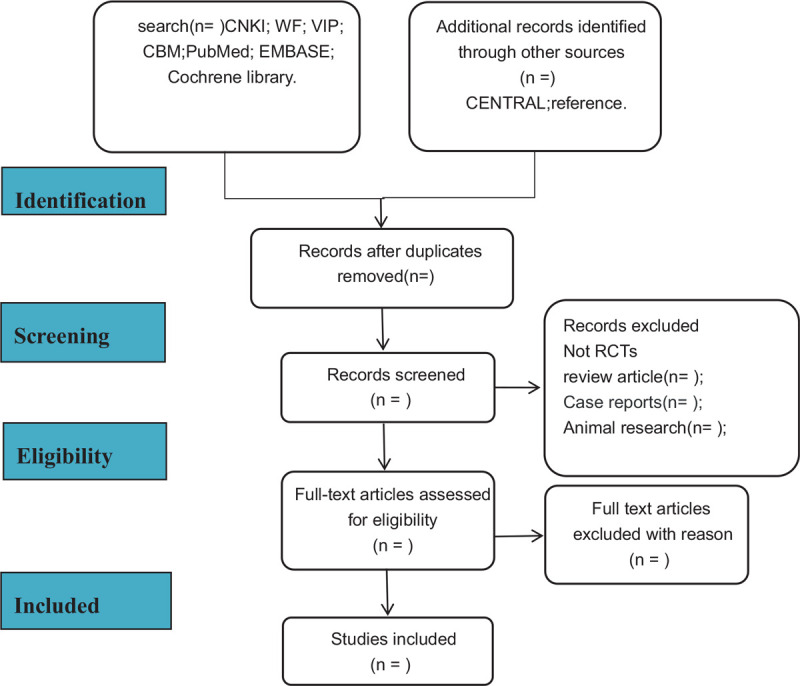
Flowchart of literature selection.

For this operation, there will be two reviewers (*Sha OuYang, DaoCheng Zhu*) strictly screened the literature and cross-checked the information individually. If they have a disagreement, consult the third reviewer (*Wei Xu*) for negotiation. The included literature process is shown in Figure 1.

#### 2.5.2 Information extraction

There are two researchers (*Sha OuYang, DaoCheng Zhu*) extracted information and input it into word 2010 independently. Then, the two researchers cross-checked these information. If there are any doubts, they must contact the third evaluator (*Wei Xu*) negotiate processing to ensure the accuracy of the information. The extracted information includes:title, author, year of publication, sample size, intervention measures and treatment course, etc. If some important information is missing in the articles, we should contact the author of these articles by phone or email.

#### 2.5.3 Methodological quality evaluation

We will use the Cochrane Reviewer's Handbook 5.0 to evaluate the quality of the final selected literature and assess the risk of bias.^[18]^ The main contents are:Random method; allocation concealment; implementation of blind method; blindness of the outcome rater; completeness of the result data; selective reporting of results; other biases. Each of the above items contains “yes,” “no” and “unclea.” The two researchers (*Sha OuYang, DaoCheng Zhu*) need to evaluate the options that meet the conditions. If there is any dispute among them, they can discuss and deal with the third evaluator (*Xu Wei*).

### 2.6 Data synthesis

#### 2.6.1 Quantitative data analysis

Enumeration data will be represented by odds ratio and 95% confidence interval, measurement data are represented by weighted mean difference and 95% confidence interval, or standardized mean difference should be used when the units were not unified.

#### 2.6.2 Heterogeneity analysis

The *I*^2^ test will be used to perform heterogeneity test. And the fixed effects model should be used when *P* > .1 and *I*^2^ < 50%; Otherwise, choose random effects model. Sensitivity analysis will be used if the heterogeneity is large. If there is substantial heterogeneity, it can be analyzed descriptively. Use Review Manager 5.4.0 line inverted funnel chart to qualitatively analyze publication bias.

#### 2.6.3 The publication bias

If there is more than 10 articles remained, Review Manager 5.4.0 line inverted funnel chart will be used to qualitatively analyze the publication bias. The graph will show the approximate shape which represents the publication bias.

#### 2.6.4 Subgroup analyses

Subgroup analysis will be implemented based on different control measures if there is a large heterogeneity.

#### 2.6.5 Sensitivity analysis

STATA 14.0 software will be used for sensitivity analysis, and the sensitivity analysis is performed to assess the reliability of the meta-analysis.

## 3 Discussion

This study is the first review of the current modern literature on electroacupuncture in treating PD, the purpose of this review is to explore more advanced evidence for electroacupuncture in treating PD. Based on the discussion part of this article, it will be described in the following aspects:

1. The function of electroacupuncture;
2. Mechanism of electroacupuncture for PD;
3. Horizontal comparison with other treatment methods or viewpoints;
4. Interpret the results;
5. Conclusion;

Electroacupuncture therapy is refer to a micro-electric current runs through the acupuncture needles after the needles stick into the acupoints to treat diseases, this therapy combine the traditional Chinese acupuncture with modern technology.^[19]^ At present, electroacupuncture has been used to regulate many systems in our body, such as nervous system, cardiovascular system, immune system, endocrine system, digestive system, etc.^[20]^ However, the mechanism of electroacupuncture in PD remains unclear. A animal study have shown that the content of tyrosine hydroxylase in substantia nigra will increase and the content of α-synuclein will decrease in PD model rats when electroacupuncture “Fengfu” and “Taichong” point, which can improve the behavioral performance of PD rats.^[21]^ Another study shown that high frequency stimulation of electroacupuncture may improve the impaired motor function of PD rats by increasing the inhibitory output of γ-aminobutyric acid (γ-GABA) in basal ganglia region.^[22]^ Although a clinical study have shown that head electroacupuncture can significantly improve the motor symptoms of PD patients, and significantly reduce the dosage of levodopa drugs.^[23]^ However, there is still a lack of systematic reviews of electroacupuncture for PD. Therefore, this study is systematically review the effectiveness and safety of electroacupuncture in treating PD, in order to provide a more reliable evidence for the treatment of PD. Certainly, this study still has some limitations:firstly, the quality of the included articles is not high, which will affect the assessment results; Secondly, there may be an inability to contact the author of articles, resulting in incomplete data results. Therefore, more high-quality randomized controlled trial and research mechanisms are needed to confirm its effectiveness, in order to objectively evaluate the effectiveness and safety of electroacupuncture in PD.

## Author contributions

**Data curation**: Wei Xu.

**Formal analysis:** Sha OuYang, DaoCheng Zhu.

**Investigation:** Zhenhai Chi, ZhiQin Wang.

**Methodology:** RiXin Chen, SiWei Duan.

**Project administration**: GenPing Zhong.

**Software:** FengTing Zhang, DaoCheng Zhu.

**Supervision**: Lin Jiao.

**Validation**: Lin Jiao.

**Visualization**: Lin Jiao.

**Writing – original draft**: DaoCheng Zhu, Wei Xu.

**Writing – review & editing:** Sha OuYang, Zhenhai ChiZhiQin Wang DaoCheng Zhu, RiXin Chen, GenPing Zhong, FengTing Zhang, GuiQin Zhou.
